# Gut microbiota and risk of endocarditis: a bidirectional Mendelian randomization study

**DOI:** 10.3389/fmicb.2024.1320095

**Published:** 2024-01-17

**Authors:** Mengyue Yang, Wen Bi, Zhijie Zhang

**Affiliations:** ^1^Department of Cardiology, Huazhong University of Science and Technology Union Shenzhen Hospital, Shenzhen, China; ^2^Department of Sports Medicine, Huazhong University of Science and Technology Union Shenzhen Hospital, Shenzhen, China

**Keywords:** gut microbiota, endocarditis, Mendelian randomization, causal relationship, probiotic

## Abstract

**Background:**

The associations between gut microbiota and cardiovascular disease have been reported in previous studies. However, the relationship between gut microbiota and endocarditis remains unclear.

**Methods:**

A bidirectional Mendelian randomization (MR) study was performed to detect the association between gut microbiota and endocarditis. Inverse variance weighted (IVW) method was considered the main result. Simultaneously, heterogeneity and pleiotropy tests were conducted.

**Results:**

Our study suggests that family Victivallaceae (*p* = 0.020), genus *Eubacterium fissicatena* group (*p* = 0.047), genus Escherichia Shigella (*p* = 0.024), genus Peptococcus (*p* = 0.028) and genus Sellimonas (*p* = 0.005) play protective roles in endocarditis. Two microbial taxa, including genus Blautia (*p* = 0.006) and genus Ruminococcus2 (*p* = 0.024) increase the risk of endocarditis. At the same time, endocarditis has a negative effect on genus *Eubacterium fissicatena* group (*p* = 0.048). Besides, no heterogeneity or pleiotropy was found in this study.

**Conclusion:**

Our study emphasized the certain role of specific gut microbiota in patients with endocarditis and clarified the negative effect of endocarditis on gut microbiota.

## 1 Introduction

Endocarditis is defined as an infectious disease of the cardiac endothelium, mainly including infection of native or artificial heart valves (Cahill and Prendergast, 2016; Wang et al., 2018). According to the Global Burden of Disease Study 2019, the incidence and mortality rates of endocarditis are 13.8 and 0.9 per 100,000 population, respectively, and the disability-adjusted life years (DALYs) are 21.9 per 100,000 population (Momtazmanesh et al., 2022). In the United States, hospitalizations for endocarditis increased from $1.58 billion to $2.34 billion from 2003 to 2016 (Alkhouli et al., 2020). As is shown in previous studies, endocarditis causes a huge burden on society. Prevention is always more important than cure, and primary prevention is an important step in disease management (Cahill et al., 2017), which highlights the importance of studying the etiology of endocarditis.

Gut microbiota live in the gastrointestinal tract and in the main coexist harmoniously with humans (Adak and Khan, 2018). Bacteria are the predominant component in the gut microbiome, but viruses, fungi, and archaea are also present and influence gut and systemic metabolism. Scientists have confirmed that gut microbes are closely related to human health by regulating metabolism and immune function (Jandhyala, 2015). Further, a number of studies have disclosed that gut microbiota may contribute to the development of neurological, metabolic, cardiovascular, and other systemic diseases (Chen et al., 2021). Currently, the role of gut microbiota in cardiovascular disease has caught attention. Gut microbiota has been proven to play an important role in coronary atherosclerosis (Jie et al., 2017), hypertension (Karbach et al., 2016), heart failure (Beale et al., 2021) and myocardial hypertrophy (Zhao M. et al., 2022) by modulating metabolites or inflammation. There is also a close relationship between gut microbiota and endocarditis. As a member of gut microbiota, *Enterococcus faecalis* has been widely reported as a risk factor for endocarditis (Ch’ng et al., 2018). It promotes the risk of endocarditis by producing virulence factors (Farman et al., 2019). *Streptococcus gallolyticus* is an intestinal commensal bacterium that promotes the risk of endocarditis in the elderly by producing gallocin (Kambarev et al., 2018; Harrington et al., 2021). Although endocarditis caused by lactic acid bacteria is rare, the mortality rate is as high as 30% (Kothari et al., 2019). In addition, endocarditis is essentially a kind of bloodstream infection, and numerous studies have confirmed that gut microbiota may play a protective role in it. Studies pointed out that Barnesiellaceae, Desulfovibrio, Butyricimonas Akkermansia, and Lachnospiraceae (Montassier et al., 2016; Yu et al., 2021) play protective roles in bloodstream infection by reducing inflammation, thus reducing the risk of endocarditis.

In recent years, more and more research has revealed the close relationship between gut microbiota and disease. As a result, fecal transplantation, probiotics, and phage therapy have been applied in clinical practice. However, the specific mechanisms, safety, and long-term effects of these treatments are still unclear. The above treatments are facing certain challenges (Ooijevaar et al., 2019; Suez et al., 2019; Łobocka et al., 2021). Gut microbiota may be a new therapeutic target for endocarditis, and it is necessary to study the causal relationship between gut microbiota and endocarditis.

Randomized controlled trials (RCTs) are considered to provide the highest level of evidence for causality in clinical research (Zabor et al., 2020). However, due to the limited human and financial resources, most of the clinical studies are observational and cannot eliminate reverse causality or confounding factors. This problem can be properly solved by MR analysis. Single nucleotide polymorphisms (SNPs) are randomly assigned during meiosis and are used as instrumental variables in MR analysis (Sekula et al., 2016). Thus, confounding factors and reverse causation are avoided in MR studies. Due to its scientific rigor and affordability, MR analysis has seen increased usage in studying cardiovascular disorders in recent years (van Oort et al., 2020; Ai et al., 2021).

In this study, we employed the MR method to reveal the relationship between gut microbiota and endocarditis. Ultimately, seven gut microbes were detected to be causally related to endocarditis.

## 2 Materials and methods

### 2.1 Study design

**Figure 1:**
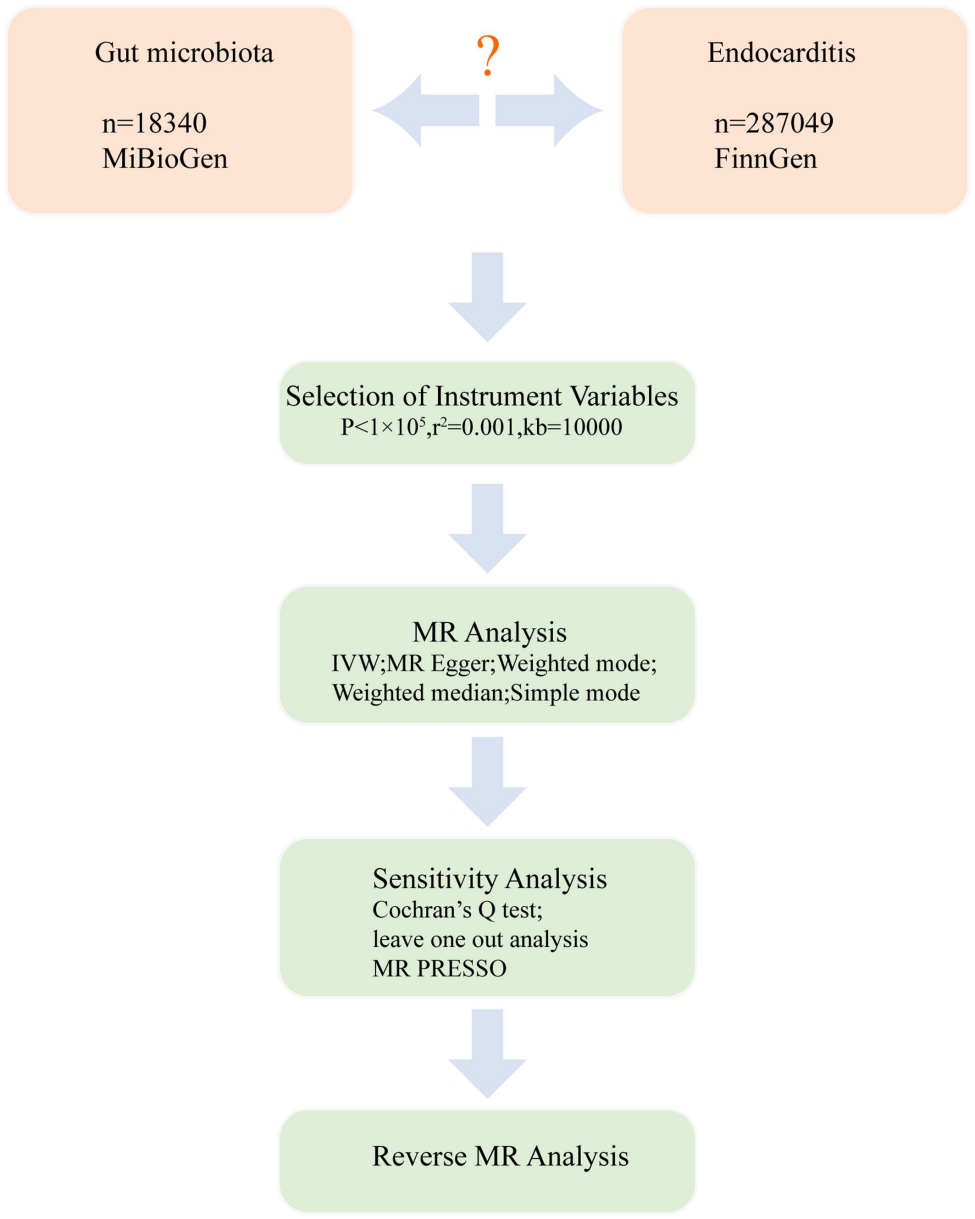
Study design. An overview of the study design.

**Figure 2:**
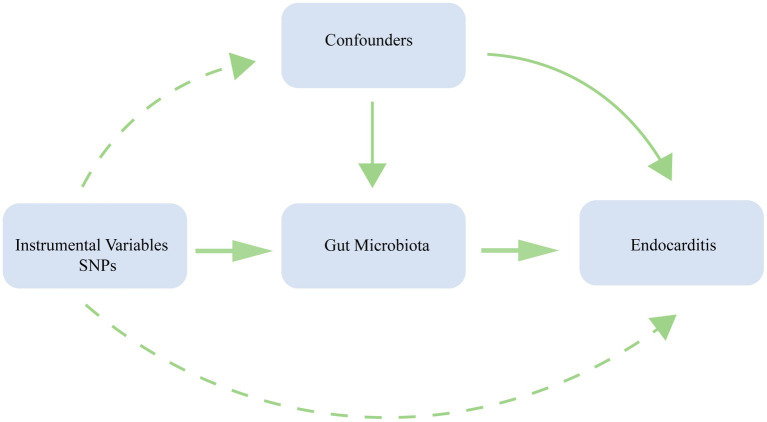
Three assumptions of MR analysis. SNP, single nucleotide polymorphisms.

We conducted a bidirectional MR analysis using data from the genome-wide association study (GWAS) to establish the causal link between gut microbiota and endocarditis. Figure 1 exhibits the study’s schematic diagram. The following three assumptions (Emdin et al., 2017) are met by this study: (1) The link between exposure and instrumental variables is considerable; (2) Instrumental variables and confounding factors are unrelated; and (3) instrumental variables only affect outcomes through exposure (Figure 2). To guarantee the validity and reliability of this study, we complied with the STROBE-MR (Strengthening the reporting of observational research in epidemiology using MR) principles (Supplementary Table S1) (Skrivankova et al., 2021).

### 2.2 Data sources

For gut microbiota, GWAS data was collected from the MiBioGen consortium.^1^ It is the largest study of human gut microbiota, and it included 18,340 individuals from 24 cohorts of multiple ancestries, about 78% of whom were European (Kurilshikov et al., 2021). 196 taxa were used for MR analysis after excluding 15 unknown taxa. For endocarditis, the GWAS data comes from the ninth version of the FinnGen,^2^ and the samples were collected by a nationwide network of Finnish biobanks (Kurki et al., 2023). By using ICD10 and ICD9 diagnosis codes, acute endocarditis, subacute endocarditis and endocarditis, valve unspecified were selected as cases. Up to 11th May 2023, it included 940 cases and 286,109 controls. There is no overlap between samples from the gut microbiota and endocarditis, thus avoiding overfitting bias. All the GWAS data is publicly available, and the original studies received ethical approval. No additional ethical approval is required.

### 2.3 Instrumental variables selection

196 species of bacteria were investigated in this study and can be sorted into five categories: phyla, class, order, family, and genus. The steps for selecting instrumental variables are: (1) SNPs strongly associated with exposure were selected, with a significance threshold of *p* < 1 × 10^−5^. In the study of Sanna et al. (2019), this threshold is considered to be the optimal threshold because it can lead to a larger variance explained. At the same time, this threshold has been widely used in previous studies (Chen et al., 2022; Yu et al., 2023). (2) As the linkage imbalance will lead to bias in MR analysis, it is necessary to remove the linkage imbalance and ensure the independence of SNPs. The parameters are set as follows: r^2^ = 0.001 and kb = 10,000. (3) When SNPs in the outcome are missing, we need to find proxy SNPs and use proxy SNPs with LD r2 > 0.8. If no proxy SNP is found, the SNP is discarded. (4) SNPs of exposure and outcome were harmonized, and the palindromic sequences were removed. (5) To avoid the association between instrumental variables and confounding factors, we removed SNPs that might be related to confounding factors, and the above process was performed using PhenoScanner (Staley et al., 2016).^3^ MR analysis was performed after these SNPs were removed. (6) To avoid weak instrumental variables, the F statistics were calculated. The formula for calculating F is R^2^ (n-k-1)/k (1-R^2^). When *F* > 10, weak instrumental variables were considered not to exist (Pierce et al., 2011).

### 2.4 MR analysis and sensitivity analysis

A MR analysis was conducted to determine the causal effect of gut microbiota on endocarditis. There were five methods: IVW, MR Egger, weighted median, simple mode, and weighted mode. Among them, IVW is the main method, and the others were applied as supplementary methods (Slob and Burgess, 2020). A fixed-effect meta-analysis model is used in the IVW method, which incorporates the causal effects of each SNP. IVW has high confidence and is used as the main method (Burgess et al., 2013). MR Egger is employed to detect whether there is pleiotropy and correct the bias (Bowden et al., 2015). The weighted median is supplemented by MR Egger, and robust results are still obtained even if 50% of the instrumental variables are invalid (Bowden et al., 2016). Simple mode and weighted mode are less effective at detecting causality but reduce the probability of Type I errors (Hartwig et al., 2017). In summary, we mainly consider the results of IVW, and the other four methods serve as references. When *p* < 0.05, we believe that exposure is significantly associated with outcome, and this significance threshold has been widely used in previous MR studies on gut microbiota (Luo et al., 2023; Song et al., 2023). Finally, we calculated power using an online site,^4^ and power > 0.8 was considered appropriate (Verbanck et al., 2018). The primary results were shown as a circle heatmap, which was drawn using the website https://www.chiplot.online/.

Finally, sensitivity analyses were implemented to avoid bias caused by heterogeneity and pleiotropy (Hemani et al., 2018). In order to determine if there was heterogeneity in this study, we employed Cochran’s Q test. Besides, the MR Egger intercept test was applied to determine if there was horizontal pleiotropy. In addition, MR-PRESSO analysis was used to find and eliminate outliers (Verbanck et al., 2018). Finally, leave-one-out analysis was carried out to determine whether there were outliers.

### 2.5 Reverse MR analysis

To detect the reverse causality between gut microbiota and endocarditis, we conducted a reverse MR analysis. With endocarditis as an exposure and gut microbiota as an outcome, a two-sample MR analysis was performed again. The methods used and the three assumptions followed were the same as described earlier.

### 2.6 Statistical software

All statistical works were implemented using the twoSampleMR package (v 0.5.6) in R software (v 4.2.1). The analysis process was performed in July 2023.

## 3 Results

### 3.1 Instrumental variables

According to the method mentioned earlier, we obtained a series of instrumental variables, including 2,142 SNPs, as shown in Supplementary Table S2. All SNPs satisfied the following conditions: (1) SNPs had a substantial correlation with exposure and *p*<1*10^−5^. (2) Confounding factors were not connected to SNPs. No SNPs associated with confounding factors were discovered by using the PhenoScanner. (3) The MR PRESSO test did not reveal any outliers (*p* > 0.05). (4) F statistics were determined, and they were all more than 10, indicating that no weak instrumental variables existed.

We found the genes where the instrumental variables (SNPs) were located according to rsID, as shown in Supplementary Table S3. Functional enrichment analysis was performed on these genes, as shown in Supplementary Figures S1, S2. The results of the Gene Ontology (GO) analysis reveal that the main functions of these genes are dendrite development and cell junction assembly. The results of Kyoto Encyclopedia of Genes and Genomes (KEGG) show that these genes are associated with Circadian entrainment and Glutamatergic synapse. The instrumental variable (SNPs) in this study is enriched in the above function or pathway, thereby indirectly increasing or reducing the risk of endocarditis by affecting gut microbiota.

### 3.2 Causal effects of gut microbiota on endocarditis

**Figure 3:**
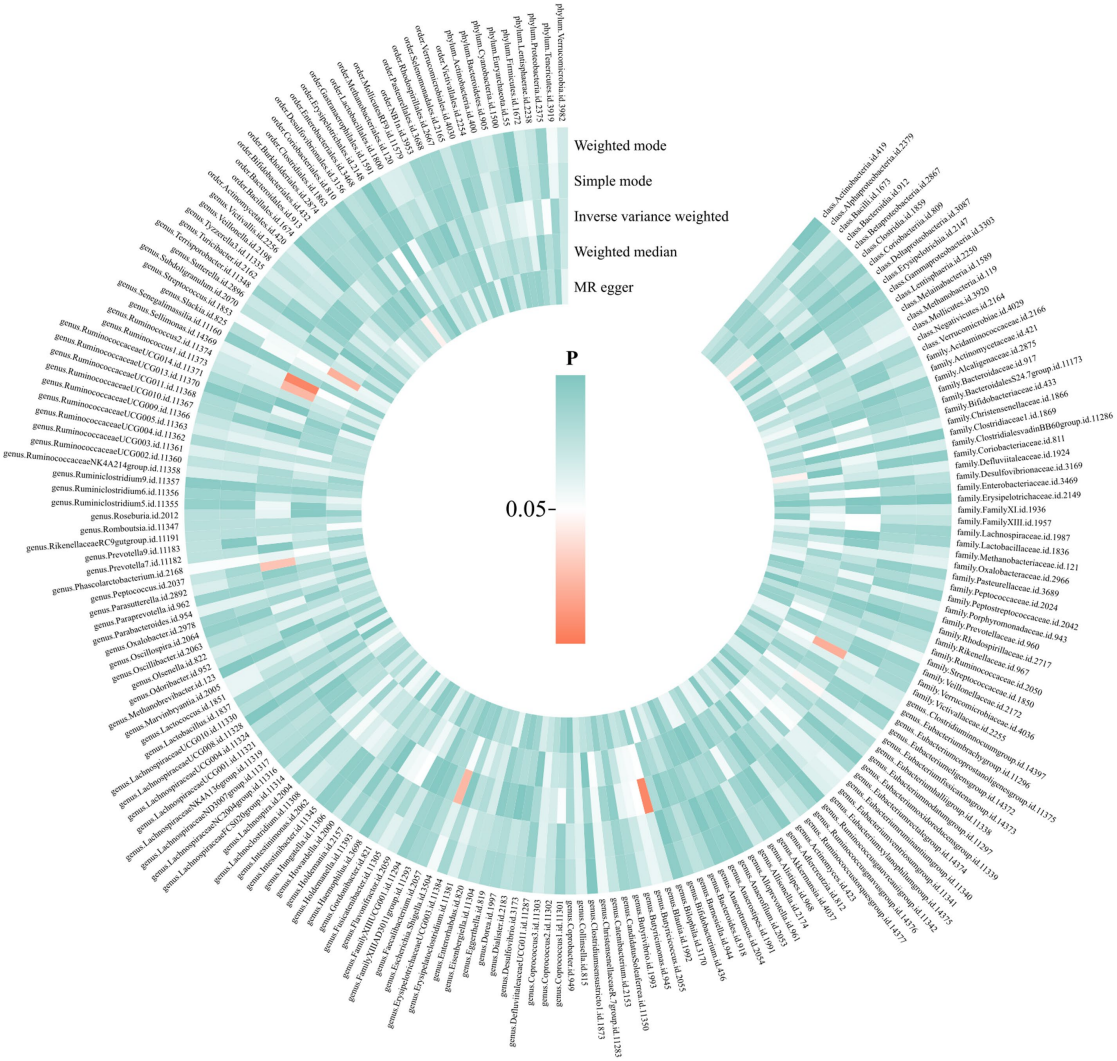
Causal effects of gut microbiota on endocarditis. From the inner circle to the outer circle, different statistical methods are represented: MR Egger, weighted median, inverse variance weighted, simple mode, and weighted mode.

**Figure 4:**
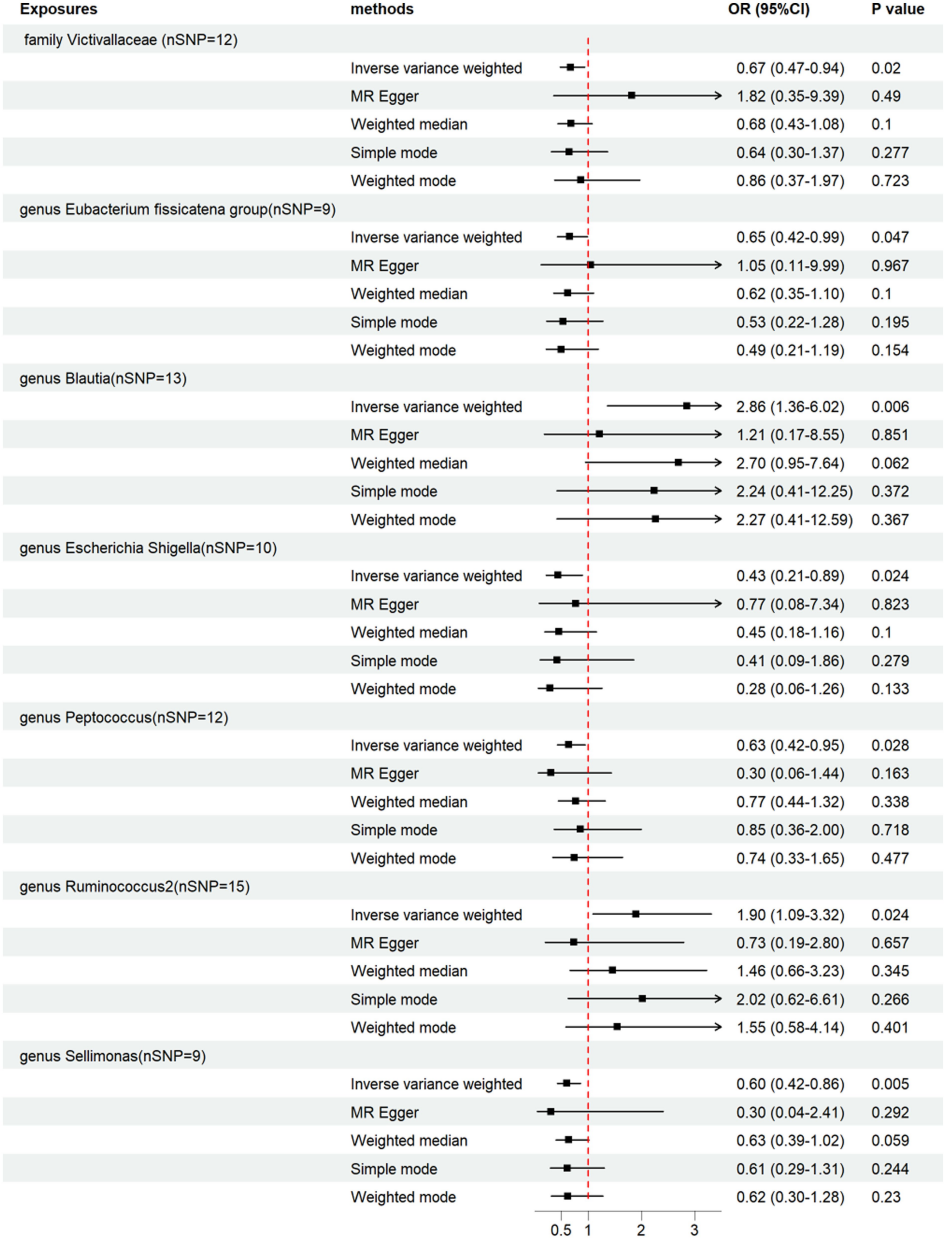
Forest plot of the causal effects of gut microbiota on endocarditis. OR, odds ratio.

**Figure 5:**
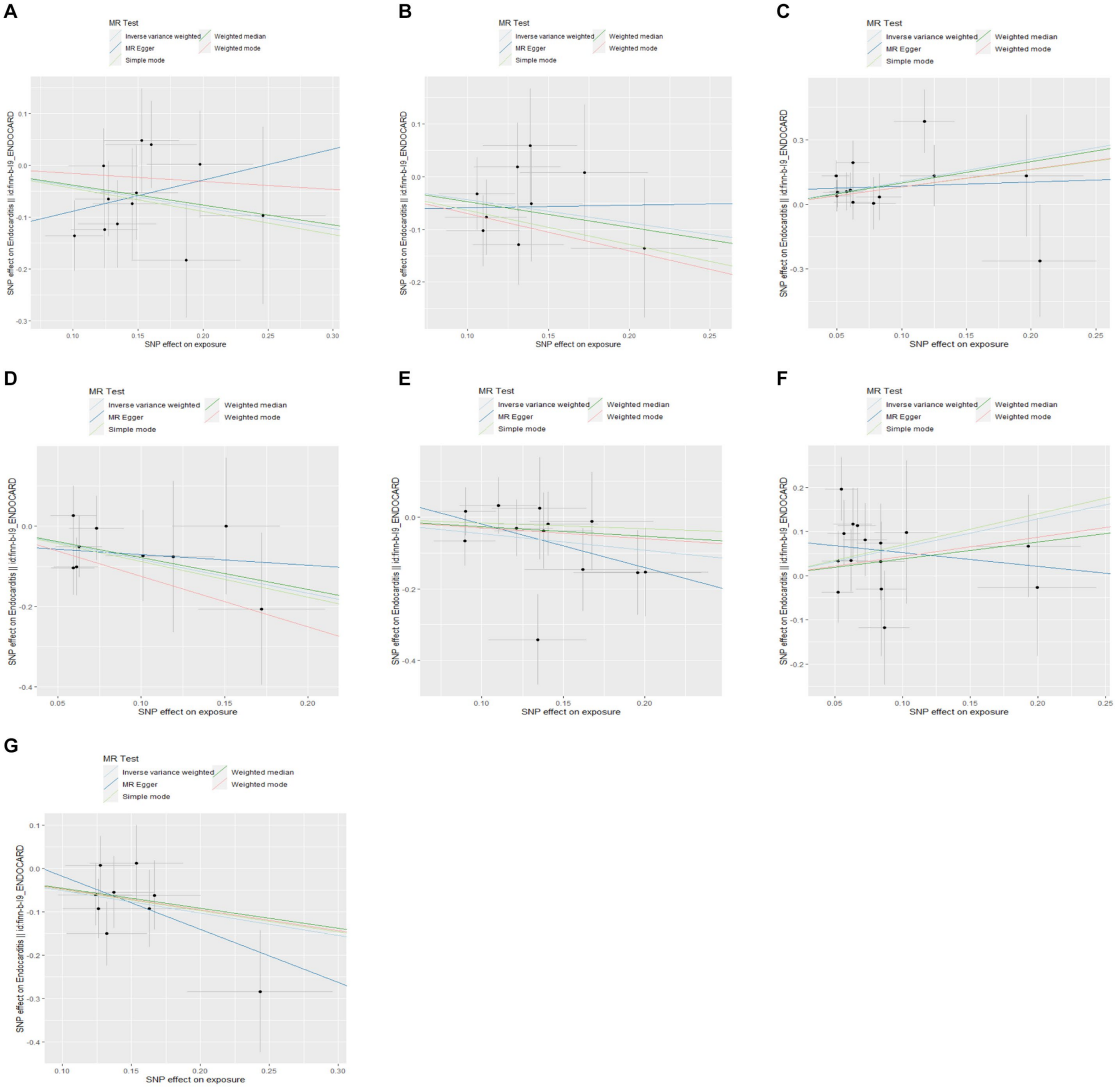
Scatter plots of the causal effects of gut microbiota on endocarditis. **(A)** Family Victivallaceae; **(B)** genus *Eubacterium fissicatena* group; **(C)** genus Blautia; **(D)** genus Escherichia Shigella; **(E)** genus Peptococcus; **(F)** genus Ruminococcus2; and **(G)** genus Sellimonas.

We performed a two-sample MR analysis of gut microbiota and endocarditis using the TwoSampleMR Package. Five methods were used and IVW methods were considered the main method. *p* < 0.05 was considered statistically significant. Seven microbial taxa were identified to have causal relationships with endocarditis. The initial analysis results are shown in Figure 3 and Supplementary Table S4. Five microbial taxa have a protective effect on endocarditis, and two increase the risk of endocarditis, as shown in Figures 4, 5. The microbial taxa that play protective roles in endocarditis are as follows: family Victivallaceae (OR: 0.67, 95%CI: 0.47–0.94, *p* = 0.020), genus *Eubacterium fissicatena* group (OR: 0.65, 95%CI: 0.42–0.99, *p* = 0.047), genus Escherichia Shigella (OR: 0.43, 95%CI: 0.21–0.89, *p* = 0.024), genus Peptococcus (OR: 0.63, 95%CI: 0.42–0.95, *p* = 0.028) and genus Sellimonas (OR: 0.60, 95%CI: 0.42–0.86, *p* = 0.005). Microbial taxa that increase the risk of endocarditis are genus Blautia (OR: 2.86, 95%CI: 1.36–6.02, *p* = 0.006) and genus Ruminococcus2 (OR: 1.90, 95%CI: 1.09–3.32, *p* = 0.024).

In addition, we conducted a sensitivity analysis, as shown in Table 1. No heterogeneity was found by using Cochran’s Q test (*p* > 0.05), and horizontal pleiotropy was not found by using the MR-Egger intercept, which indicates the reliability of the above results. Finally, we carried out visualization. Funnel plots and the leave-one-out method were used. As shown in Supplementary Figures S3, S4, the funnel plot is symmetrical, and no outliers in the SNPs are found. The leave-one-out method also shows that removing a single SNP does not have a fundamental effect on the overall results.

**Table 1:** Result of heterogeneity and pleiotropy test.

Gut microbes	Heterogeneity			MR-PRESSO		Pleiotropy	
	Method	Q	Q_Pval	MR-PRESSO outlier-corrected	MR_PRESSO global test Pval	MR egger_intercept	*p*
Family Victivallaceae	IVW	8.224	0.693	NA	0.759	−0.148	0.247
	MR egger	6.712	0.752				
genus *Eubacterium fissicatena* group	IVW	4.307	0.828	NA	0.842	−0.064	0.679
	MR egger	4.121	0.766				
genus Blautia	IVW	10.768	0.549	NA	0.561	0.066	0.371
	MR egger	9.898	0.540				
genus Escherichia Shigella	IVW	3.929	0.916	NA	0.444	−0.045	0.614
	MR egger	3.654	0.887				
genus Peptococcus	IVW	8.787	0.642	NA	0.654	0.102	0.355
	MR egger	7.848	0.644				
genus Ruminococcus2	IVW	11.953	0.610	NA	0.667	0.084	0.150
	MR egger	9.611	0.725				
genus Sellimonas	IVW	5.085	0.748	NA	0.831	0.103	0.526
	MR egger	4.640	0.704				

### 3.3 Reverse MR analysis

To find out if there is reverse causality, we performed reverse MR analysis on the above 7 microbial taxa, taking endocarditis as exposure and gut microbiota as the outcome. We obtained nine instrumental variables, as shown in Supplementary Table S5, and we also calculated F statistics, which were greater than 10, avoiding the bias caused by weak instrumental variables. Similarly, we mainly consider the IVW results. The results of reverse MR analysis are shown in Supplementary Table S6. We found that there is a reverse causality relationship between the genus *Eubacterium fissicatena* group (OR = 0.940, 95%CI: 0.88–0.99, *p* = 0.048) and endocarditis. Additionally, no horizontal pleiotropy or heterogeneity were found either.

## 4 Discussion

In this study, we examined the potential causal connection between gut microbiota and endocarditis using the largest GWAS data on gut microbiota. It is the first time to study the connection between endocarditis and gut microbiota by using MR analysis. Five bacterial taxa, including family Victivallaceae, genus *Eubacterium fissicatena* group, genus Escherichia Shigella, genus Peptococcus and genus Sellimonas may play protective roles in the pathogenesis of endocarditis. Two bacterial taxa, including genus Blautia and genus Ruminococcus2, are risk factors for endocarditis. It provides new ideas for the diagnosis and treatment of endocarditis. Furthermore, a negative causal relationship between endocarditis and genus *Eubacterium fissicatena* group was confirmed.

Human health and illness are influenced by gut microbiota. Gut microbiota plays an important role in neurological diseases (Qiu et al., 2022), inflammatory bowel disease (Qiu et al., 2022), respiratory diseases (Ma et al., 2022), tumors (Xu et al., 2021) and cardiovascular diseases (Jin et al., 2019). In this study, we focused on the relationship between gut microbiota and cardiovascular disease. Studies have found that gut microbiota promotes or reduces the risk of cardiovascular disease by regulating metabolites (Sun et al., 2021) and inflammatory responses (Shikata et al., 2019; Wu et al., 2022). As the gut-heart axis has been proposed recently (Chen et al., 2020; Zhao P. et al., 2022), the link between gut microbiota and endocarditis is receiving more attention. It is reasonable to speculate that gut microbiota is related to endocarditis, and our study confirms the causal relationship between them.

Previous studies on the relationship between gut microbiota and endocarditis are very limited. The mechanism by which gut microbiota promotes or alleviates endocarditis is still unclear.

Our study identified that five bacterial taxa including family Victivallaceae, genus *Eubacterium fissicatena* group, genus Escherichia Shigella, genus Peptococcus and genus Sellimonas reduce the risk of endocarditis, and previous studies support our findings to some extent. Genus *Eubacterium fissicatena* group produces butyrate, which not only maintains normal intestinal permeability, but also exerts anti-inflammatory effects (Cammann et al., 2023). Genus Peptococcus plays an important role in antioxidant and maintenance of normal intestinal morphology (Zhang L. et al., 2021; Zhu et al., 2022). Besides, genus Sellimonas may play an important role in maintaining intestinal homeostasis by regulating metabolites (Munoz et al., 2020). However, studies on the probiotic function of family Victivallaceae and genus Escherichia Shigella are very limited. The mechanisms mentioned above might explain the probiotic functions of gut microbiota in endocarditis to some extent. Further, the five protective bacteria identified in this study may be used to prevent endocarditis through probiotic supplementation or fecal transplantation.

At the same time, our study also found that two bacterial taxa, including genus Blautia and genus Ruminococcus2, promote the risk of endocarditis. Genus Blautia may promote inflammatory responses by increasing the production of IL-6 and TNF-α (Wei et al., 2022). Ruminococcus2 decreases the expression of zonula occludens-1 and mucin 2, thereby damaging the intestinal barrier and promoting disease (Zhou et al., 2020). These mechanisms mentioned above may explain why these two bacteria promote endocarditis. Further, the concentration of genus Blautia and genus Ruminococcus2 in stool may serve as an indicator to predict the risk of endocarditis.

However, gut microbiota includes not only bacteria but also fungi and viruses (Fragkou et al., 2021). It is necessary to discuss the role of fungi and viruses in endocarditis. Previous studies have shown that gut fungi and viruses play an important role in endocarditis (Tattevin et al., 2014; Ezzatpour et al., 2023). The main components of gut fungi are Candida, Saccharomycetales, and Aspergillus, etc. (Zhang et al., 2022). Besides, Candida and Aspergillus are the main causes of fungal endocarditis (Antinori et al., 2014). They first cause fungemia, which leads to the adhesion of fungi to the heart valves, thus leading to fungal endocarditis (Ammannaya and Sripad, 2019). The gut virome mainly includes bacteriophages and eukaryotic viruses (Bhagchandani et al., 2023), and most of which are bacteriophages. Bacteriophages can enter the systemic circulation and play an important role in pro-inflammatory and anti-inflammatory responses, thereby playing a positive or negative role in human health (Neil and Cadwell, 2018; Stockdale and Hill, 2021). We can reasonably speculate that gut virome may also play an important role in promoting or alleviating endocarditis. Finally, gut mycobiome and virome may lead to changes in the structure of gut bacteria or intestinal mucosal permeability, which may cause the host to be susceptible to certain opportunistic pathogens or metabolites, leading to infectious diseases or noninfectious disease (Zhang F. et al., 2021; Cao et al., 2022).

Certain strengths are worth mentioning in our study: First, the MR method was implemented to analyze the causal link between gut microbiota and endocarditis, avoiding confounding factors and reverse causality. Secondly, compared with randomized controlled trials, MR studies save time and effort and can provide possible ideas for RCT design. Lastly, this study used the largest GWAS database on gut microbiota, which makes our study reliable.

However, there are some limitations. First of all, the data on gut microbiota and endocarditis are from European populations, and the generalization of our conclusions in other populations has certain limitations. Secondly, while our work sheds light on a potential causal relationship between gut microbiota and endocarditis, additional research on the precise mechanism is required. Mendelian randomization does not explain the underlying biological mechanisms of the causality between diseases, just as classical epidemiological studies do. Basic experiments are needed to discover the specific mechanism. Last but not least, the gut microbiota includes bacteria, fungi, viruses, and other microorganisms. Our study only analyzes the role of gut bacteria in endocarditis without analyzing the influence of gut fungi, viruses, and other microorganisms on endocarditis, which makes this study have certain limitations to some extent.

## 5 Conclusion

In conclusion, our work reveals the causal relationship between gut microbiota and endocarditis, and it provides a target for the diagnosis and treatment of endocarditis. The application of probiotic supplements or fecal transplantation may be used for the prevention of endocarditis.

## Data availability statement

The datasets presented in this study can be found in online repositories. The names of the repository/repositories and accession number(s) can be found in the article/Supplementary material.

## Author contributions

MY: Writing – original draft, Formal analysis, Software. WB: Writing – review & editing. ZZ: Supervision, Writing – review & editing.

## Footnotes

^1^https://mibiogen.gcc.rug.nl/

^2^https://r9.risteys.finngen.fi/

^3^http://www.phenoscanner.medschl.cam.ac.uk/

^4^https://shiny.cnsgenomics.com/mRnd/
