# Supplementary material for: Gut microbiota and risk of endocarditis: a bidirectional Mendelian randomization study
Source: Front Microbiol. 2024 Jan 17;15:1320095. doi: 10.3389/fmicb.2024.1320095 (PMC10827985; doi:10.3389/fmicb.2024.1320095)
Supplement: Supplementary file 1 [file Data_Sheet_1.docx]

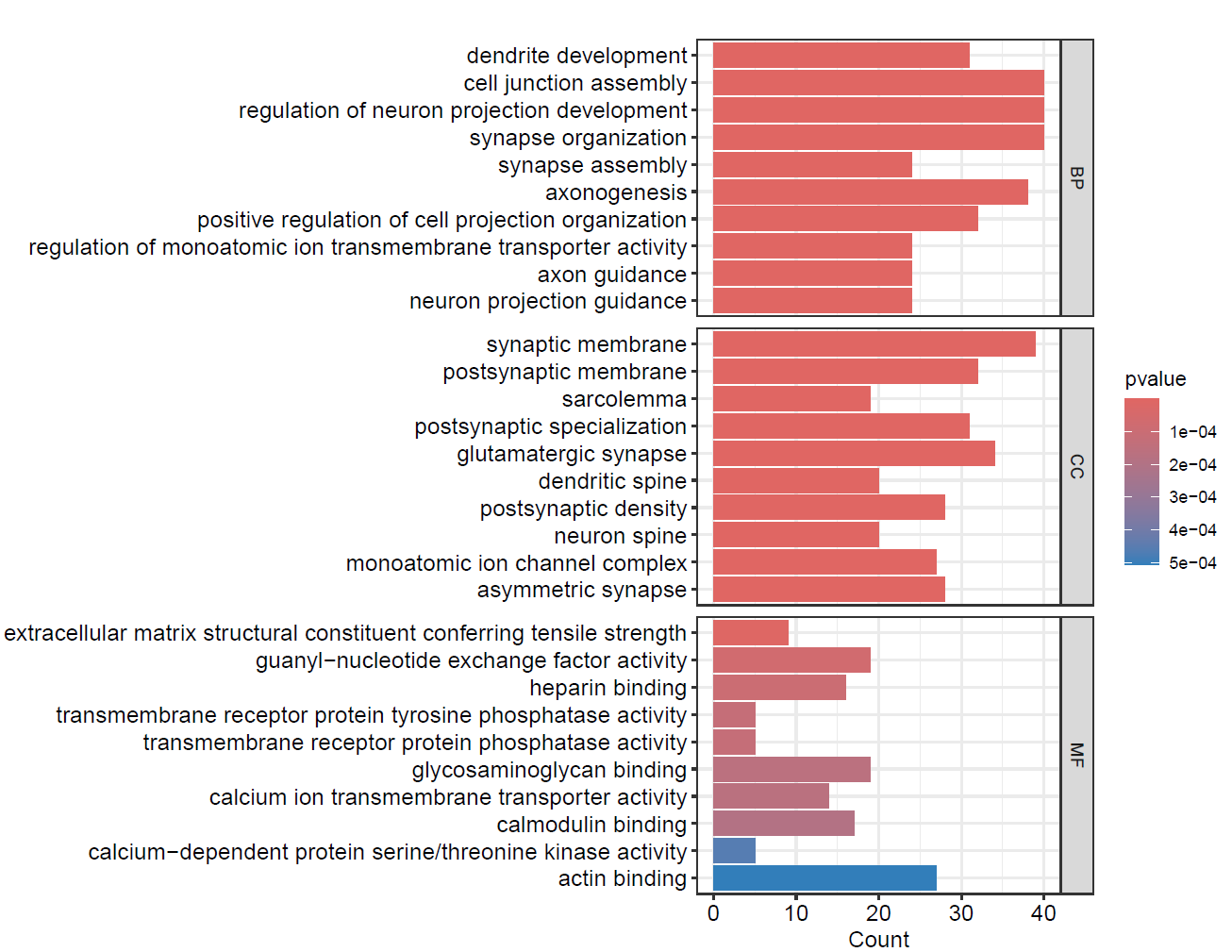


Figure S1 Gene Ontology (GO) analysis of the genes where SNPs are located.


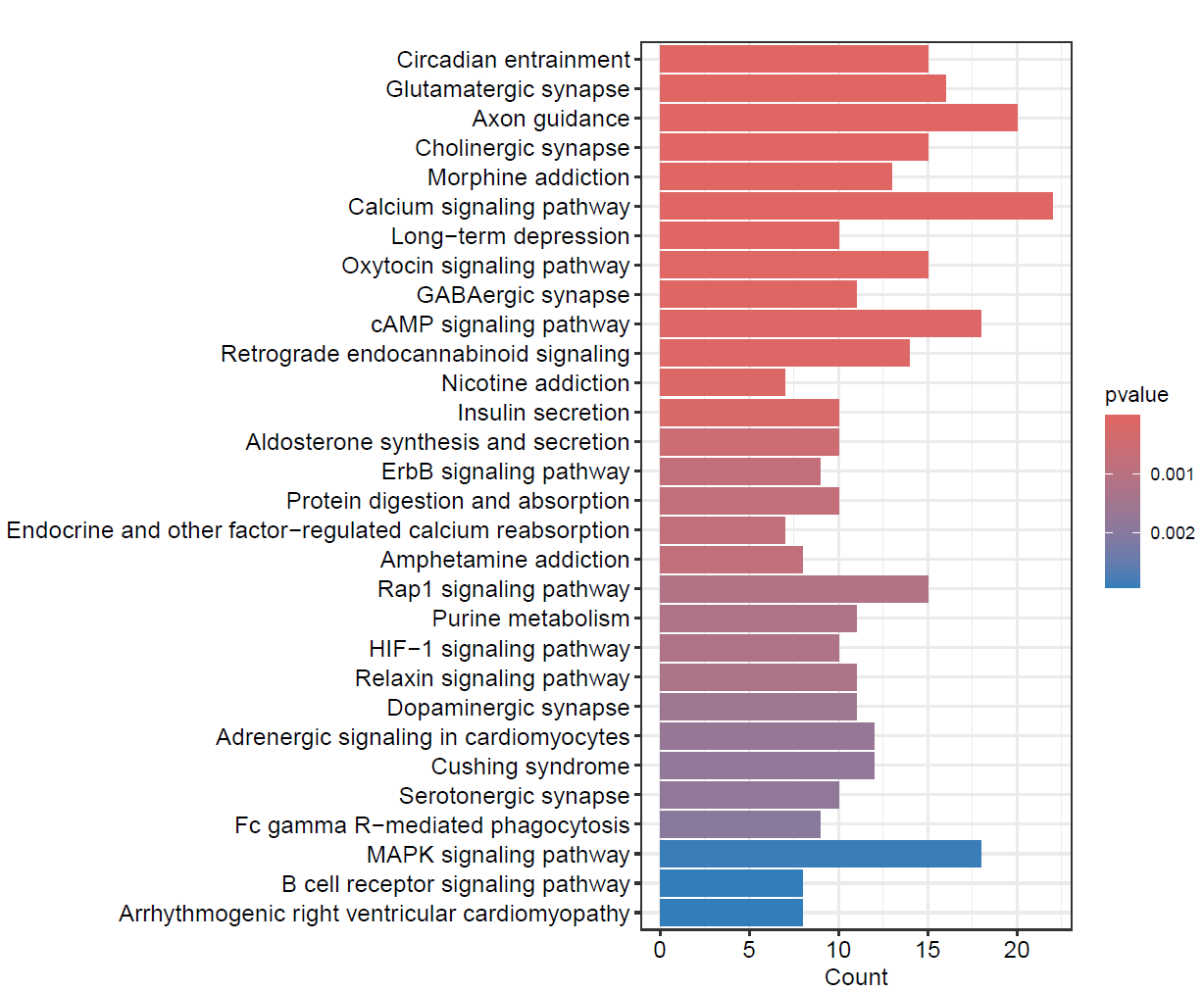


Figure S2 Kyoto Encyclopedia of Genes and Genomes (KEGG) analysis of the genes where SNPs are located.


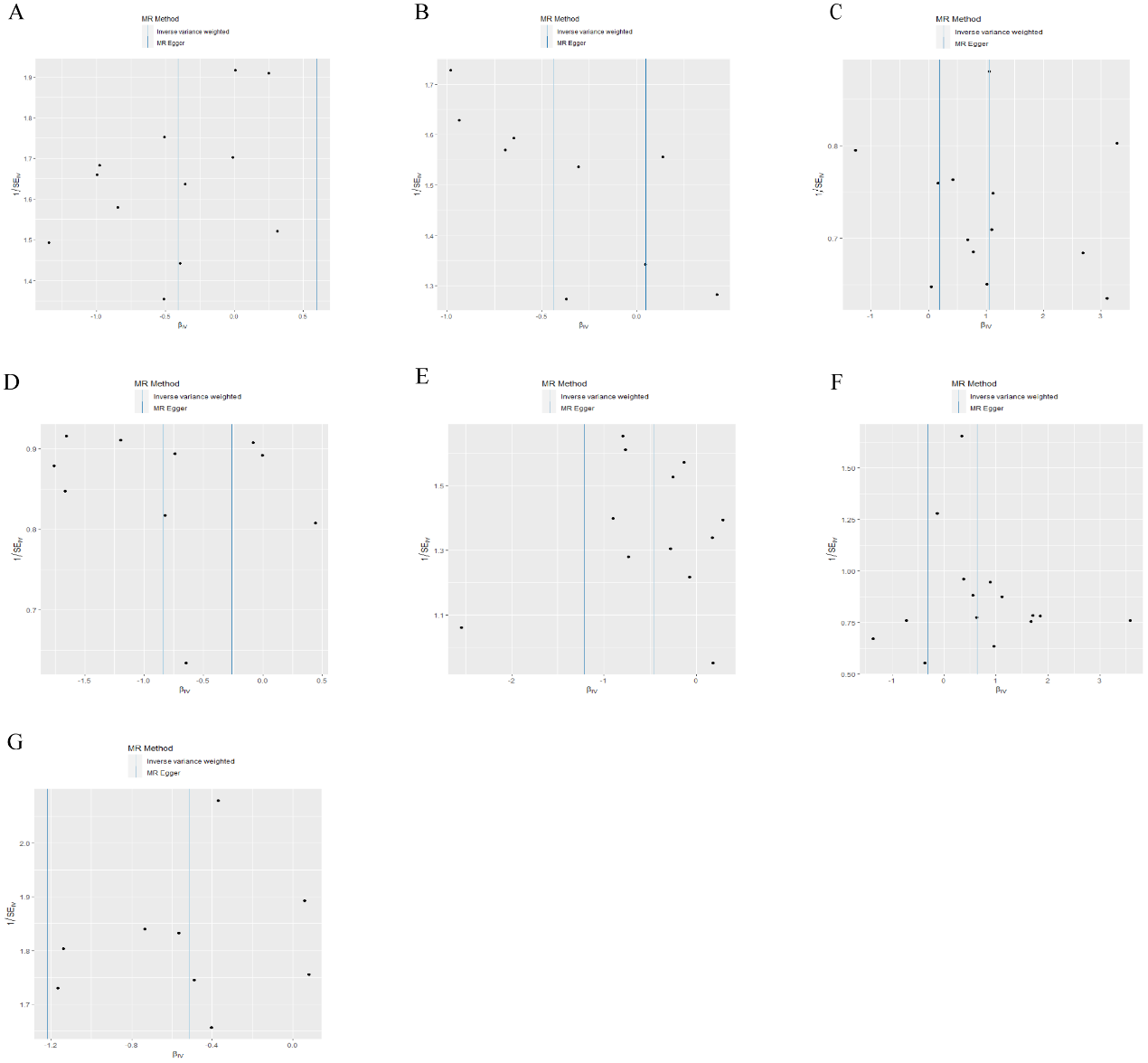


Figure S3 : Funnel plots. A. family Victivallaceae ; B. genus Eubacterium fissicatena group; C. genus Blautia; D. genus Escherichia Shigella; E. genus Peptococcus ; F.genus Ruminococcus2; G. genus Sellimonas.


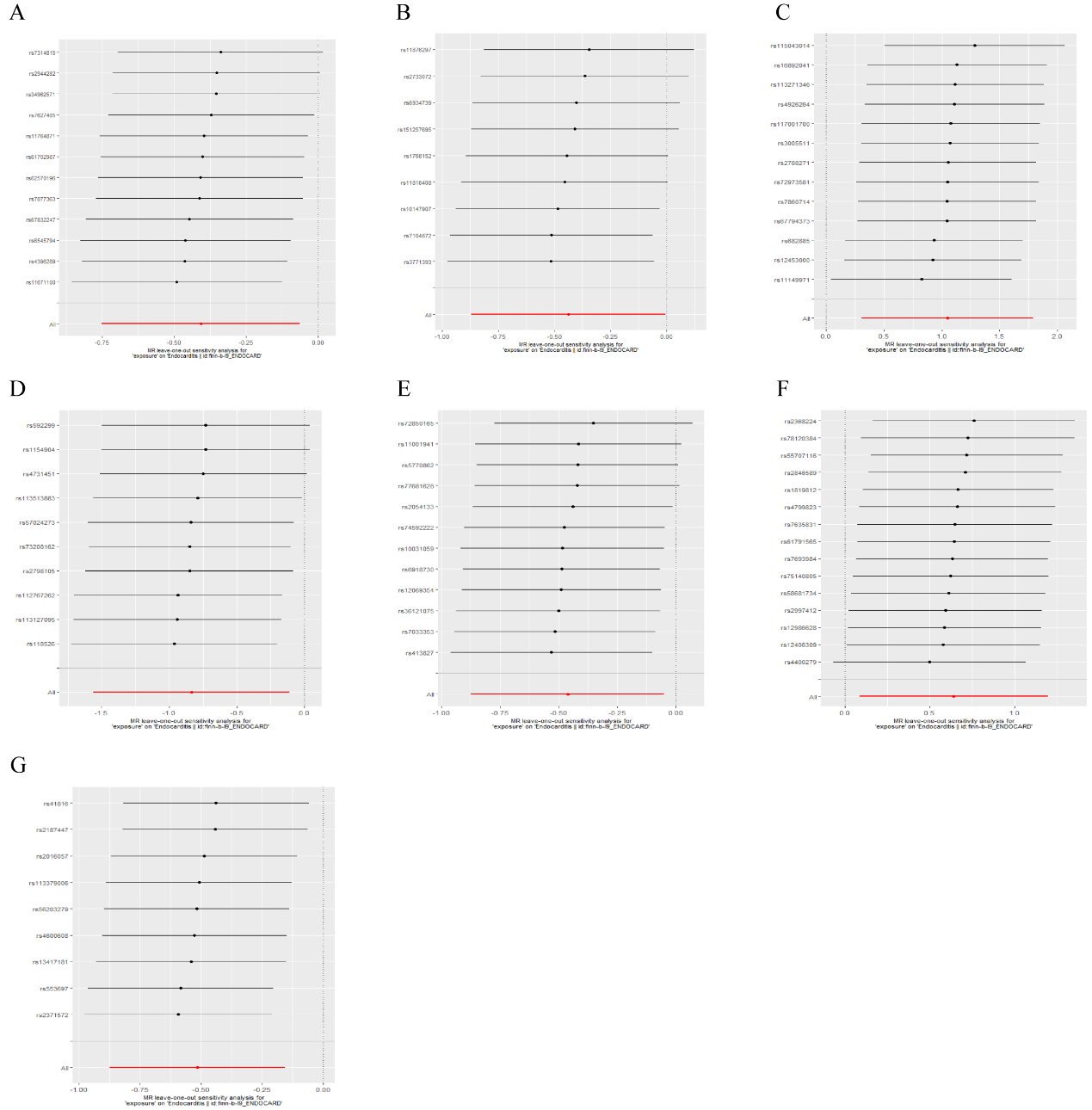


Figure S4: Leave-one-out sensitivity analyses of the causal effects of gut microbiota on endocarditis A. family Victivallaceae ; B. genus Eubacterium fissicatena group; C. genus Blautia; D. genus Escherichia Shigella; E. genus Peptococcus ; F.genus Ruminococcus2; G. genus Sellimonas.
